# Obestatin regulates cardiovascular function and promotes cardioprotection through the nitric oxide pathway

**DOI:** 10.1111/jcmm.13277

**Published:** 2017-07-26

**Authors:** Claudia Penna, Francesca Tullio, Saveria Femminò, Carmine Rocca, Tommaso Angelone, Maria C. Cerra, Maria Pia Gallo, Iacopo Gesmundo, Alessandro Fanciulli, Maria Felice Brizzi, Pasquale Pagliaro, Giuseppe Alloatti, Riccarda Granata

**Affiliations:** ^1^ Department of Clinical and Biological Sciences University of Turin Turin Italy; ^2^ National Institute of Cardiovascular Research Bologna Italy; ^3^ Department of Biology, Ecology and E.S. University of Calabria Rende CS Italy; ^4^ Department of Life Sciences and Systems Biology University of Turin Turin Italy; ^5^ Department of Medical Sciences University of Turin Turin Italy

**Keywords:** obestatin, myocardial contractility, coronary flow, cardioprotection, ischaemia/reperfusion, post‐conditioning

## Abstract

Patients with ischaemic heart disease or chronic heart failure show altered levels of obestatin, suggesting a role for this peptide in human heart function. We have previously demonstrated that GH secretagogues and the ghrelin gene‐derived peptides, including obestatin, exert cardiovascular effects by modulating cardiac inotropism and vascular tone, and reducing cell death and contractile dysfunction in hearts subjected to ischaemia/reperfusion (I/R), through the Akt/nitric oxide (NO) pathway. However, the mechanisms underlying the cardiac actions of obestatin remain largely unknown. Thus, we suggested that obestatin‐induced activation of PI3K/Akt/NO and PKG signalling is implicated in protection of the myocardium when challenged by adrenergic, endothelinergic or I/R stress. We show that obestatin exerts an inhibitory tone on the performance of rat papillary muscle in both basal conditions and under β‐adrenergic overstimulation, through endothelial‐dependent NO/cGMP/PKG signalling. This pathway was also involved in the vasodilator effect of the peptide, used both alone and under stress induced by endothelin‐1. Moreover, when infused during early reperfusion, obestatin reduced infarct size in isolated I/R rat hearts, through an NO/PKG pathway, comprising ROS/PKC signalling, and converging on mitochondrial ATP‐sensitive potassium [mitoK(ATP)] channels. Overall, our results suggest that obestatin regulates cardiovascular function in stress conditions and induces cardioprotection by mechanisms dependent on activation of an NO/soluble guanylate cyclase (sGC)/PKG pathway. In fact, obestatin counteracts exaggerated β‐adrenergic and endothelin‐1 activity, relevant factors in heart failure, suggesting multiple positive effects of the peptide, including the lowering of cardiac afterload, thus representing a potential candidate in pharmacological post‐conditioning.

## Introduction

Several studies have suggested an important role for the growth hormone (GH)‐releasing peptides, such as ghrelin and GH secretagogues (GHS), or the ghrelin gene‐derived product obestatin, as homeostatic regulators of the cardiovascular system 1, 2, 3, 4. These peptides have been shown to modulate cardiac inotropism 5, 6, 7, 8 and vascular tone 9, 10, 11, 12, 13, 14, and to reduce cell death and contractile dysfunction in hearts subjected to ischaemia/reperfusion (I/R) 15, 16, 17.

Obestatin may play a role in cardiac function in humans, in both physiological and pathological conditions 18. Indeed, obestatin levels were slightly increased in saliva of overweight patients with ischaemic heart disease 19, while they were reduced in serum of patients with type 2 diabetes mellitus 20 and obesity 21, two pathological conditions associated with cardiovascular diseases. The levels of both ghrelin and obestatin were found increased in patients with chronic heart failure and cachexia 22 as well as in spontaneously hypertensive rats (SHR) 23. Yet, recent studies showed that obestatin may exert beneficial cardiovascular effects in humans, by producing vascular relaxation through activation of endothelium‐dependent nitric oxide (NO) signalling 24. The link between obestatin and NO has been confirmed in obese subject, where obestatin induced vascular relaxation via specific activation of endothelium‐dependent NO signalling 25.

We have previously shown that pre‐treatment of isolated rat hearts with obestatin reduced infarct size and contractile dysfunction induced by I/R. In addition, obestatin exerted a similar preconditioning‐like effect in rat H9c2 cells and isolated ventricular myocytes, displaying an antiapoptotic effect when these cells were subjected to I/R. In these two preconditioning models, the protective effect of obestatin was due to activation of protein kinase C (PKC), phosphoinositide 3‐kinase (PI3K) or extracellular signal‐regulated kinase (ERK)1/2 pathways 15. Despite the above observations, to date, the physiological role of obestatin in stress conditions is still largely unknown and its cardiovascular effects need to be studied further.

As PI3K may lead to NOS activation *via* Akt phosphorylation, it can be argued that NO plays a role also in the regulation of cardiac function and in cardioprotection by obestatin. Our hypothesis is that the activation of PI3K/Akt/NO and protein kinase G (PKG) pathway by obestatin is involved in protecting the heart when challenged by either adrenergic, endothelinergic or I/R stress. To test this hypothesis, obestatin‐induced modulation of adrenergic or endothelinergic response in non‐ischaemic conditions was studied in rat papillary muscle and whole heart, respectively. Moreover, the protective effect of obestatin against I/R injury was assessed in post‐ischaemic Langendorff rat heart. As in post‐conditioning the PI3K/Akt/NO and PKG pathways converge on mitochondrial K(ATP) [mito K(ATP)] channels 26, we also verified the involvement of mitoK(ATP) in this context.

## Materials and methods

### Reagents

Rat obestatin was purchased from Tocris (Bristol, UK). The chemicals were purchased from Sigma‐Aldrich (St. Louis, MO, USA). The reagents to assess myocardial infarction were provided by Merck (Darmstadt, Germany). Antibodies for Phospho‐eNOS (Catalog #9571), eNOS (Catalog #9572) and PKG‐1 (Catalog #13511s) were from Cell Signaling Technology (Euroclone, Milan, Italy); antibody for actin was from Santa Cruz Biotechnology (DBA, Milan, Italy), (Catalog #sc‐376421).

### Animals

Male Wistar rats (*n* = 80; body weight 450–550 g, age 3–4 months) received human care in compliance with the Italian law. All procedures were performed according to institutional guidelines in compliance with the Italian law (DL‐116, Jan. 27, 1992 and D.L. N.26, 04/03/2014) and International law and policies (new directive 2010/63/EU). The protocol was approved by the Ethical Committee of the University of Turin and by the Italian Ministry of Health.

### Isolated papillary muscle

Papillary muscles were dissected free from the left ventricle under a stereomicroscope, superfused with oxygenated Tyrode solution (composition, in mM: 154 NaCl, four KCl, two CaCl_2_, one MgCl_2_, 5.5 d‐glucose and five HEPES; pH adjusted to 7.35 with NaOH) at 37°C, and driven at constant frequency (120 beats/min.) with a pair of electrodes connected to a stimulator (302 T Anapulse; W. P. Instruments, New Haven, CT, USA) *via* a stimulus isolator (model 305‐R; W. P. Instruments) operating in constant‐current mode. Isometric twitches were evaluated by a transducer (model 60‐2997; Harvard Instruments) and continuously acquired and recorded by a PowerMac computer using Labview software (National Instruments, Italy) 27. Before each experiment, papillary muscles were equilibrated in oxygenated (100% O_2_) Tyrode solution for 30 min. In preliminary experiments, we tested the effects of different concentrations of obestatin (25, 50 and 100 nM) in basal experimental condition. The minimal concentration of obestatin (Tocris) giving a significant inotropic effect (50 nM, see *Results*) was chosen to study its antiadrenergic effect. To this purpose, the positive inotropic effect induced by β‐adrenergic stimulation (isoproterenol, ISO, 100 nM) was compared to that exerted by ISO in the presence of 50 nM obestatin on the same preparation (*n* = 5). Each treatment lasted 10 min., then the perfusion was switched to Tyrode solution alone to study the reversibility of the effect. NG‐nitro‐l‐arginine methyl ester (L‐NNA, 1 mM) was used to block NO synthesis, Wortmannin (WN, 100 nM) to block PI3K activity and 1H‐[1,2,4] oxadiazole‐[4,4‐a] quinoxalin‐1‐one (ODQ, 10 μM) to block guanylate cyclase (GC) activity (in each case, *n* = 5). Five experiments were performed in the presence of (D‐Lys3)‐GHRP‐6 (1 μM), a selective antagonist of the ghrelin receptor GH GHS receptor type 1a (GHSR‐1a) 28, 29. To investigate the role of endocardial endothelium (EE) in the action of obestatin, additional experiments (*n* = 5) were performed in papillary muscles treated with 0.5% Triton X‐100 for 1‐2 sec. to remove EE, followed by 20–30 min. washout with Tyrode solution 30. All drug‐containing solutions were prepared fresh before the experiments. The basic advantage of the papillary muscle is to avoid influences of flow and/or beating rate variation on determining inotropic response (Gregg or garden hose effect) 31.

Papillary muscles were dissected free from the left ventricle under a stereomicroscope, superfused with oxygenated Tyrode solution (composition, in mM: 154 NaCl, four KCl, two CaCl_2_, one MgCl_2_, 5.5 d‐glucose and five HEPES; pH adjusted to 7.35 with NaOH) at 37°C, and driven at constant frequency (120 beats/min.) with a pair of electrodes connected to a stimulator (302 T Anapulse; W. P. Instruments, New Haven, CT, USA) *via* a stimulus isolator (model 305‐R; W. P. Instruments) operating in constant‐current mode. Isometric twitches were evaluated by a transducer (model 60‐2997; Harvard Instruments) and continuously acquired and recorded by a PowerMac computer using Labview software (National Instruments, Italy) 27. Before each experiment, papillary muscles were equilibrated in oxygenated (100% O_2_) Tyrode solution for 30 min. In preliminary experiments, we tested the effects of different concentrations of obestatin (25, 50 and 100 nM) in basal experimental condition. The minimal concentration of obestatin (Tocris) giving a significant inotropic effect (50 nM, see *Results*) was chosen to study its antiadrenergic effect. To this purpose, the positive inotropic effect induced by β‐adrenergic stimulation (isoproterenol, ISO, 100 nM) was compared to that exerted by ISO in the presence of 50 nM obestatin on the same preparation (*n* = 5). Each treatment lasted 10 min., then the perfusion was switched to Tyrode solution alone to study the reversibility of the effect. NG‐nitro‐l‐arginine methyl ester (L‐NNA, 1 mM) was used to block NO synthesis, Wortmannin (WN, 100 nM) to block PI3K activity and 1H‐[1,2,4] oxadiazole‐[4,4‐a] quinoxalin‐1‐one (ODQ, 10 μM) to block guanylate cyclase (GC) activity (in each case, *n* = 5). Five experiments were performed in the presence of (D‐Lys3)‐GHRP‐6 (1 μM), a selective antagonist of the ghrelin receptor GH GHS receptor type 1a (GHSR‐1a) 28, 29. To investigate the role of endocardial endothelium (EE) in the action of obestatin, additional experiments (*n* = 5) were performed in papillary muscles treated with 0.5% Triton X‐100 for 1‐2 sec. to remove EE, followed by 20–30 min. washout with Tyrode solution 30. All drug‐containing solutions were prepared fresh before the experiments. The basic advantage of the papillary muscle is to avoid influences of flow and/or beating rate variation on determining inotropic response (Gregg or garden hose effect) 31.

### Isolated perfused heart

The methods were similar to those previously described 32, 33, 34, 35. In brief, animals were anaesthetized and treated for 10 min. with heparin. After killing, the chest was opened and the heart rapidly excised. Isolated rat hearts were weighed, attached to the perfusion apparatus and retrogradely perfused with oxygenated Krebs‐Henseleit (KH) buffer (127 mM NaCl, 17.7 mM NaHCO_3_, 5.1 mM KCl, 1.5 mM CaCl_2_, 1.26 mM MgCl_2_, 11 mM d‐glucose), gassed with 95% O_2_ and 5% CO_2_. A small hole in the left ventricular wall was performed to allow drainage of the Thebesian flow, and a polyvinyl‐chloride balloon was placed into the left ventricle. Left ventricular pressure, coronary flow and perfusion pressure were continuously monitored to assess the conditions of the preparation. The hearts were electrically paced at 280 bpm and kept in to a temperature controlled chamber (37°C). Each heart was allowed to stabilize for 40 min., after stabilization the preparation was switched to constant pressure or constant flow perfusion, at which time baseline parameters were recorded 33, 34, 36.

### Coronary flow studies at constant pressure perfusion

The effects of obestatin on coronary flow were studied on isolated hearts perfused at constant pressure (80 mm Hg) both in basal conditions and under stimulation with 1 nM endothelin‐1 (ET‐1). The basic advantage of the constant pressure is to avoid influences of pressure variation on determining vessel tone (myogenic or stress relaxation responses). The role of NO and PI3K in the vasodilator effect of obestatin was tested using the specific inhibitors L‐NNA (1 mM) and Wortmannin (WN, 100 nM), respectively (in each case, *n* = 5) 37.

### Ischaemia and reperfusion experiments at constant flow (CF) perfusion

The cardioprotective effects of obestatin were investigated on isolated hearts perfused at constant flow. The basic advantage of the constant flow perfusion is to avoid influences of flow variation in determining reperfusion injury. CF was adjusted with a proper pump to obtain a typical coronary perfusion pressure of 80–85 mm Hg during the initial part of stabilization. Thereafter, the same flow level (9 ± 1 ml/min./g) was maintained throughout the experiment.

### I/R experimental protocols

**Figure 1:**
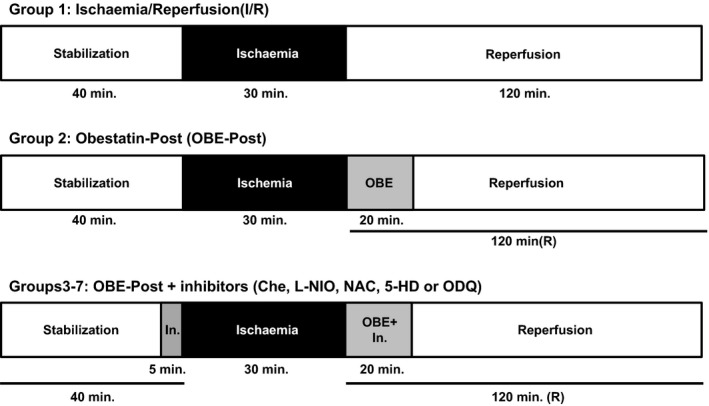
Experimental protocols. Before ischaemia, hearts were randomly allocated to one of the experimental groups. All the hearts underwent 40 min. of stabilization, 30 min. of ischaemia and 120 min. of reperfusion, for a total of 190 min. Group 1 hearts (I/R, Group 1, *n* = 6) were exposed to I/R protocol only. Group 2 hearts were treated with obestatin (75 nM) during the initial 20 min. of reperfusion (OBE‐Post group; *n* = 9). Group 3–7 hearts were treated with obestatin and different specific inhibitors of NO/ROS/PKCε/mitoK(ATP) channel/GC pathway (Che 1 μM; L‐NIO 1 μM; NAC 10 μM; 5‐HD 10 μM; ODQ 10 μM; *n* = 5 for each group). The inhibitors (In.) were administered during the last 5 min. of stabilization and the initial 20 min. of reperfusion.

After the stabilization period, hearts were subjected to a specific protocol, which included in all groups a 30 min. of global no‐flow ischaemia. In all groups, the 30 min. of ischaemia were followed by a period of 120 min. of reperfusion (see below). Pacing was discontinued on initiation of ischaemia and restarted after the third minute of reperfusion 33, 34. Experimental protocols for groups 1–7 are described in Figure 1. After stabilization, the hearts of the control group (I/R, Group 1, *n* = 6) were exposed I/R protocol only. In Group 2 (OBE‐Post group; *n* = 9), immediately after ischaemia the hearts were reperfused in the presence of obestatin (75 nM) for 20 min. Group 3–7 hearts were treated with obestatin and different specific inhibitors of NO/ROS/PKCε/mitoK(ATP) channel/GC pathway (Che 1 μM; L‐NIO 1 μM; NAC 10 μM; 5‐HD 10 μM; ODQ 10 μM; *n* = 5 for each group). The antagonists were administered during the last 5 min. of stabilization and the first 20 min. of reperfusion at the concentrations described previously 33, 34, 38. These concentrations of the inhibitors did not affect basal cardiac performance, as demonstrated by our preliminary results.

### Assessment of myocardial injury

Infarct size was assessed as previously described 32, 33, 34. In brief, the hearts were removed from perfusion apparatus at the end of reperfusion, and the left ventricles (LV) dissected into 2–3 mm circumferential slices. Following 20 min. of incubation at 37°C in 0.1% nitro‐blue tetrazolium in phosphate buffer solution, unstained necrotic tissue was carefully separated from stained viable tissue by an independent observer. The necrotic mass was weighted and expressed as % of total left ventricular mass. Although in this model the whole heart underwent normothermic ischaemia, only the LV had a fixed volume and preload; therefore, only the LV mass was considered as risk area.

### Western blotting

Immunoblot analysis was performed as previously described 32, 34. An additional group of hearts perfused with buffer alone for 90 min. (Sham) was added (*n* = 3). LV samples were collected after 90 minutes in Sham group, and either after 20 min. of reperfusion in I/R hearts or at the end of treatment during reperfusion (after 20 min.) for OBE‐Post. All tissues were snap frozen in liquid nitrogen before being stored at −80 °C until protein extraction. Briefly, 50 μg of total proteins for P‐eNOS and PKG‐1α were resolved in 8% and 10%, respectively, in SDS‐PAGE and incubated with the specific antibody. Blots were reprobed with antibodies against total eNOS and actin for PKG‐1α, for normalization.

### Statistical analysis

All data are expressed as means ± S.E.M. One‐Way anova and Tukey's HSD (honestly significant difference) for post‐anova comparisons were used to evaluate the statistical significance of the differences between groups. Significance was established when *P* < 0.05.

## Results

### Obestatin blunts β‐adrenergic response *via* an endothelial‐dependent PI3K‐Akt‐NO‐cGMP pathway in isolated papillary muscle

**Figure 2:**
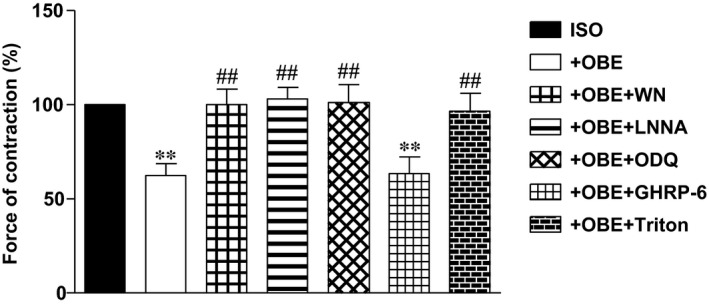
Antiadrenergic effect of obestatin in isolated rat papillary muscle. The positive inotropic effect of ISO (100 nM) is compared with that exerted by ISO in the presence of 50 nM obestatin (OBE). L‐NNA (1 mM), WN (100 nM), ODQ (10 μM) and (D‐Lys3)‐GHRP‐6 (1 μM) were used to block NO synthesis, PI3K or sGC activity, and the ghrelin receptor GHSR‐1a, respectively. Triton X‐100 (0.5%) was used to remove endocardial endothelium. Values are expressed as % of the contraction force induced by ISO alone. **P < 0.01 ISO + OBE or ISO + OBE + GHRP‐6 *versus* ISO alone; ^##^ *P* < 0.01 ISO + OBE *versus* ISO + OBE + inhibitors and Triton.

Under basal conditions, obestatin induced a biphasic effect on myocardial contractility, characterized by a slight, transient enhancement (at 50 nM: 115.0 ± 3.5% of baseline value, *n* = 5), followed by a dose‐dependent reduction of developed tension (94.9 ± 5.4, 83.8 ± 4.4 and 62.7 ± 5.3% of baseline value at 25, 50 and 100 nM, respectively). These effects were completely reversible: 20 min. washout restored contractile force to 101.8 ± 1.2% of baseline values in papillary muscle treated with 100 nM obestatin. Moreover, obestatin (50 nM) exerted a significant antiadrenergic effect, reducing by 40% the positive inotropic effect of ISO (*P* < 0.01 *versus* ISO alone) (Fig. 2). To test the role of the PI3K/Akt/NO/cGMP pathway in the antiadrenergic effect of obestatin, papillary muscles were pre‐treated with WN, L‐NNA or ODQ before the administration of ISO or ISO + OBE, to block PI3K, NO or guanylate cyclase activity, respectively. As shown in Figure 2, all these drugs abolished the antiadrenergic effect of obestatin, suggesting that the PI3K/Akt/NO/cGMP pathway plays an important role in the inotropic effects of this peptide. The positive inotropic effect of ISO alone (196.2 ± 16.8%) was not modified by the inhibitors (170.2 ± 8.7%, WN + ISO; 178.0 ± 13.9%, L‐NNA + ISO; 199.9 ± 24.4%, ODQ + ISO).

The presence of (D‐Lys3)‐GHRP‐6, a selective antagonist of ghrelin receptor GHSR‐1a, did not alter the antiadrenergic effect of obestatin, suggesting that the action of obestatin does not involve the ghrelin receptor (*P* < 0.01 *versus* ISO). Finally, to verify if NO may derive from endocardial endothelial cells, we tested whether the removal of EE would modify the antiadrenergic effect of obestatin. While removal of EE with 0.5% Triton X‐100 did not significantly modify the inotropic effect of ISO under basal conditions (203.6 ± 16.1% of baseline, *n* = 5), the antiadrenergic effect of obestatin was completely blocked (191.8 ± 12.7%, ISO + OBE).

### Obestatin blunts endothelinergic response and modulates vascular function *via* PI3K/NO pathway in isolated rat heart perfused at constant pressure

**Figure 3:**
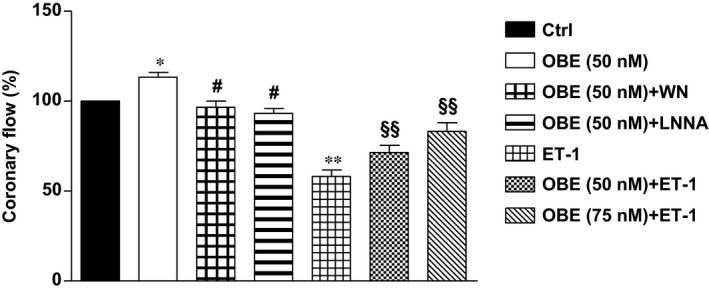
Effect of obestatin on coronary flow. The effect of 50 nM obestatin was studied in basal conditions in the isolated rat heart perfused at constant pressure. The role of NO and PI3K in the vasodilator effect of obestatin was tested using the specific inhibitors L‐NNA (1 mM) and WN (100 nM), respectively. Two different concentrations of obestatin (50 and 75 nM) were also studied under stimulation with ET‐1 (1 nM). Values are expressed as percentage of baseline coronary flow. *P < 0.05 OBE *versus* Control; **P < 0.01 ET‐1 *versus* Control; ^#^ *P* < 0.05 OBE + L‐NNA or OBE + WN *versus* OBE; ^§§^ *P* < 0.01 OBE + ET‐1 *versus* ET‐1.

In basal conditions, 50 nM obestatin exerted a moderate, but significant (*P* < 0.05 *versus* control) vasodilator effect (flow = 113.3 ± 2.8% of baseline), which was completely blocked by both L‐NNA and WN (flow = 93.2 ± 2.7 and 96.6 ± 3.5%, respectively; *n* = 5 for each group; in both cases, *P* < 0.05). Importantly, the vasoconstrictor effect of 1 nM ET‐1 (flow = 58.2 ± 3.5% of baseline) was dose dependently reduced by 50 or 75 nM obestatin (flow = 71.4 ± 4.0 and 83.2 ± 4.9% of baseline, respectively; *n* = 5 for each group; in both cases, (*P* < 0.01) (Fig. 3).

### Post‐ischaemic obestatin blunts I/R injury *via* NO‐PKG/PKC pathway and mitoK(ATP)‐ROS signalling in isolated rat heart perfused at constant flow

**Figure 4:**
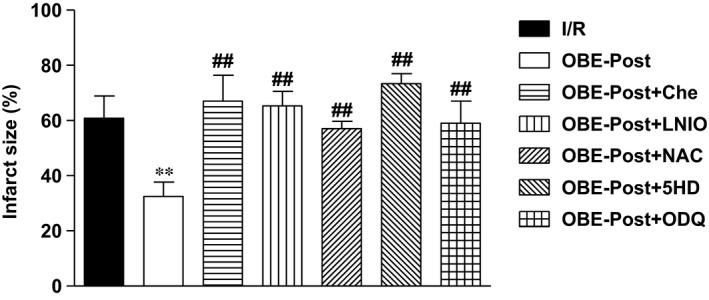
Obestatin given in the early reperfusion reduces infarct size in the isolated rat hearts subjected to 30 min. of ischaemia and 120 min. of reperfusion. Hearts were treated with vehicle alone (I/R) or with obestatin (75 nM) at the onset of reperfusion (OBE‐Post), in either absence or presence of the indicated inhibitors (see *Materials and Methods* for details). Infarct size was determined by nitro‐blue tetrazolium staining and expressed as percentage of ventricular mass. **P < 0.01 OBE‐Post *versus* I/R; ^##^ *P* < 0.01 OBE‐Post *versus* OBE‐Post + inhibitors.

The administration of obestatin (75 nM) during the early reperfusion significantly reduced cell damage observed after I/R in the isolated rat hearts. In particular, infarct size was reduced from 60 ± 8% (I/R group) to 33 ± 5% (OBE‐Post group; *P* < 0.01 *versus* I/R group) of LV mass (Fig. 4). The cardioprotective effect of obestatin was abrogated by treatment with the PKC antagonist chelerythrine (Che), the NOS inhibitor (L‐NIO), the ROS scavenger *N*‐acetyl‐l‐cysteine (NAC), the mitoK(ATP) channel blocker 5‐hydroxydecanoate (5‐HD) and the GC inhibitor (ODQ). The infarct sizes measured in these hearts were 66 ± 9%, 65 ± 11%, 57 ± 2%, 73 ± 3% and 59 ± 8%, respectively (*P* < 0.01 *versus* OBE‐Post).

### Obestatin counteracts the inhibitory effect of I/R on phosphorylation of eNOS and expression of PKG‐1α

**Figure 5:**
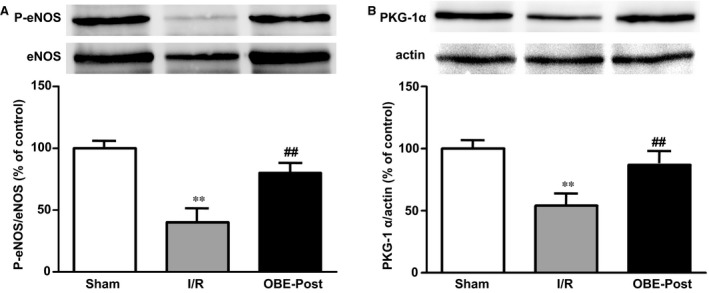
Effect of obestatin on phosphorylation of eNOS and on PKG‐1α expression. Western blot analysis was performed in lysates from LVs collected in Sham hearts after 90 minutes of buffer perfusion, and in either I/R hearts during reperfusion (after 20 min.) or in I/R reperfused with 75 nM obestatin (OBE‐Post) for 20 min. (**A**) eNOS phosphorylation and (**B**) PKG‐1α expression (upper panels). Blots, each representative of three independent experiments, were reprobed with total eNOS or actin antibody for normalization (lower panels). Graphs represent the densitometric analysis of P‐eNOS or PKG‐1α normalized to total eNOS or actin, respectively, and reported as per cent of Sham. **P < 0.01 I/R *versus* Sham; ^##^ *P* < 0.01 OBE‐Post *versus* I/R.

We next investigated the effect of obestatin on activation of eNOS/NO/PKG pathway, known to be involved in postconditioning 39. Our results show that in isolated rat hearts, I/R reduced both the phosphorylation of eNOS and the expression of PKG‐1α, with respect to Sham. However, obestatin, given at the time of early reperfusion, counteracted this effect and strongly increased both phospho‐eNOS (Fig. 5A) and PKG‐1α (Fig. 5B). These results further suggest the involvement of eNOS/NO/PKG signalling in the cardioprotective action of obestatin.

## Discussion

In the present study, we sought to unveil the role of obestatin as a regulator of cardiac function, in particular, in stress conditions. Our results show that obestatin displays an inhibitory tone on the performance of rat papillary muscle, both under basal conditions and β‐adrenergic stimulation, through an endothelial‐dependent NO/sGC/PKG pathway. This ‘antiadrenergic’ action suggests a putative counter‐regulatory role for obestatin in cardiovascular homeostasis, by protecting the heart against β‐adrenergic receptor‐induced overstimulation in conditions of stress. Furthermore, our findings suggest that the same pathway is also implicated in the ability of obestatin to modulate vascular function and to counteract the effect of endothelin‐1 on vasoconstriction. Obestatin also exerts a post‐conditioning‐like effect, by reducing the infarct size in isolated heart subjected to I/R, through activation of an NO/ROS/PKC pathway, converging on mitoK(ATP) channel. The involvement of this pathway is corroborated by evidence showing that obestatin promotes the phosphorylation of eNOS and expression of PKG‐1α, both reduced by I/R.

Obestatin is a recently discovered amidated peptide, originating from the ghrelin precursor pre‐pro‐ghrelin 18, 40. It has been suggested that both obestatin and ghrelin participate in a complex regulatory system; however, the intracellular pathways activated by obestatin and its role in physiopathological conditions are largely unknown 18, 41. Although it has been initially suggested that obestatin antagonizes the central activities of ghrelin, through interaction with the orphan receptor GPR39 40, studies from our group and others have shown that obestatin exerts peripheral actions, including those at the cardiovascular level, similar to those of acylated and unacylated ghrelin and other GHS 18. In fact, ghrelin improves cardiac function 42, displays cardioprotective effects after I/R injury 17, 43, and inhibits apoptosis in cardiomyocytes and endothelial cells through mechanisms independent of GHS‐R1a, mediated by activation of PI3K/Akt and ERK1/2 44. Likewise, obestatin displays a preconditioning‐like function against cardiac alterations induced by I/R 15 or diabetes 45. Moreover, like ghrelin, obestatin reduces apoptosis in rat H9c2 cardiac cells and isolated ventricular myocytes subjected to I/R, through PKC, PI3K/Akt or ERK1/2 pathways 15. Recently, obestatin has been shown to also attenuate doxorubicin‐induced cardiac dysfunction through inhibition of cardiomyocyte apoptosis *via* nuclear factor erythroid 2‐related factor 2 (Mhrt‐Nrf2)‐dependent pathway 46. In addition, both ghrelin and obestatin levels were found increased in chronic heart failure (CHF) patients with cachexia; however, the ghrelin to obestatin ratios were lower in these patients than in controls, suggesting that ghrelin and ratio of ghrelin to obestatin are independent predictors of the development of cardiac cachexia 22. Ghrelin and obestatin levels, as well as the ratio of ghrelin to obestatin were also higher in spontaneously hypertensive rats, suggesting a role for the ghrelin/obestatin system in the regulation of blood pressure 23.

The receptor(s) involved in the effects of obestatin has yet to be determined. In fact, the role of GPR39, the putative receptor for obestatin 40, has been questioned by different groups 18, 47, 48, although binding sites for obestatin have been shown in different cell types 15, 29, 49, 50. Our present findings have demonstrated that the selective antagonist of ghrelin receptor (D‐Lys3)‐GHRP‐6 did not alter the antiadrenergic action of obestatin, suggesting GHS‐R1a‐independent effect of the peptide and involvement of a different receptor. Interestingly, we have previously shown that the glucagon‐like peptide 1 receptor (GLP‐1R) may be implicated in the physiopathological actions of obestatin in several cell types 29, 49, 51, 52. This may also be possible for cardiomyocytes and endothelial cells, which express GLP‐1R 53, although more studies are needed to clarify this hypothesis.

Obestatin is likely to play a significant role in cardiovascular function also in humans, both under physiological and pathological conditions 18, 25. However, the dose of obestatin used in this study was several times (about 250‐fold) higher than the levels detected in biological fluids in patients with ischaemic heart disease 19. Of note, it must be taken into account that our experimental conditions are quite different from those present in patients. We studied the effect of a single acute treatment in a cardiac preparation from a healthy animal, while in patients we can suppose that the heart is exposed to chronically elevated levels of obestatin in a pathological scenario, in which a severe endothelial dysfunction and other circulating mediators are present.

The ability of obestatin to modulate cardiac contractility in basal conditions and during β‐adrenergic stimulation, confirms and extends our previous observations on the effects of the synthetic peptidyl GHS hexarelin and ghrelin with its endogenous derivatives, on mechanical tension developed by rat and guinea pig papillary muscles 5, 54. The cardioprotective action of obestatin, when infused at the early reperfusion, expands our previous observations on the positive effects of the peptide when given before an ischaemic event 15. As treatments at the onset of myocardial reperfusion are more feasible than pre‐treatments 55, 56, 57, the present results may have a greater relevance, in view of its possible clinical application.

Regarding the mechanisms of action of obestatin in reducing infarct size, we show here that the obestatin‐induced protection is abolished by either upstream antagonists, namely the broad range inhibitor of PKCs, CHE, the NOS blocker L‐NIO, as well as by the downstream target antagonists, such as the mitoK(ATP) channel blocker, 5HD or the ROS scavenger, NAC. The involvement of the NO/PKG pathway is confirmed by the co‐infusion of obestatin with specific inhibitor for NO/PKG pathway, and is supported by results on phosphorylation of eNOS and expression of PKG‐1α. Accordingly, several data demonstrate that a pathway similar to that activated by obestatin is also induced by Post‐C manoeuvres 36, 58, 59. In fact, it is known that I/R reduces the basal myocardial content of cGMP and the ability of cardiomyocytes to respond to stimulators of cGMP synthesis, and the Post‐C manoeuvres abolish these deleterious effects 36. Moreover, previous studies have demonstrated that preservation of the sGC/PKG pathway during reperfusion, using pharmacological strategies to either increase cGMP synthesis or reduce its degradation, attenuates death of cardiomyocytes 60.

Here, we also show that obestatin exerts a vasodilator action on coronary vessels, being able to counteract the potent vasoconstrictor effect of ET‐1. Moreover, the coronary dilatory effects of obestatin are due to stimulation of NOS and NO release from endothelial cells, through the PI3K/Akt/eNOS pathway. Our findings are consistent with previous data showing that obestatin induces vascular relaxation in rat aorta 13 or inhibits endothelin‐1‐induced vasoconstriction in humans 25 through activation of endothelium‐dependent NO signalling, indicating that obestatin may be important in the regulation of vascular function. Therefore, our results suggest a novel physiological function for obestatin as a cardiostatin, possibly able to play a relevant role in the natural defence against exaggerated cardiac hyperactivity. This is the case of the ‘neuroendocrine storm’, where activation of the sympathetic nervous system (SNS) during heart failure leads to imbalance of the SNS and vagal activity and to a further worsening of cardiac dysfunction 61. Of note, high endothelin‐1 levels have been associated with poor outcome after myocardial infarction 62, making our observations on the effects of obestatin particularly interesting, as this peptide may be proposed as multiple target agent able to counteract maladaptive feed‐back/responses in post‐ischaemic condition.

In conclusion, this study shows that obestatin induces cardioprotection against I/R injury and counteracts both exaggerated β‐adrenergic and endothelin‐1 activity. Therefore, our findings suggest that obestatin may play an important role in modulating cardiovascular function in conditions of stress. In particular, we show that the postconditioning‐like, antiadrenergic and anti‐endothelinergic effects are dependent on the PI3K/Akt/NO pathway. The fact that obestatin counteracts exaggerated β‐adrenergic activity, a relevant part of the neuroendocrine scenario in heart failure 63, and dilates coronary vessels *in vitro* (present study), as well as human vessels *in vivo* 25, suggests that this peptide may exert multiple positive effects. In line with these evidences, as β‐blockade is largely used as post‐ischaemic treatment, and exaggerated endothelinergic response may be a possible therapeutic target, the present work suggests that exogenous obestatin may represent a new tool for pharmacological postconditioning.

## Authors' contributions

CP, FT, SV, CR, TA, MCC, MPG and IG performed the study; CP, MFB, PP, GA and RG designed the study, analysed the data and revised the manuscript; GA and RG wrote the manuscript.

## Conflict of interest

All the authors declare no conflict of interest.
